# The Involvement of Iron in Traumatic Brain Injury and Neurodegenerative Disease

**DOI:** 10.3389/fnins.2018.00981

**Published:** 2018-12-20

**Authors:** Maria Daglas, Paul A. Adlard

**Affiliations:** The Florey Institute of Neuroscience and Mental Health, The University of Melbourne, Parkville, VIC, Australia

**Keywords:** iron, metals, traumatic brain injury, neurodegeneration, oxidative stress, inflammation

## Abstract

Traumatic brain injury (TBI) consists of acute and long-term pathophysiological sequelae that ultimately lead to cognitive and motor function deficits, with age being a critical risk factor for poorer prognosis. TBI has been recently linked to the development of neurodegenerative diseases later in life including Alzheimer’s disease, Parkinson’s disease, chronic traumatic encephalopathy, and multiple sclerosis. The accumulation of iron in the brain has been documented in a number of neurodegenerative diseases, and also in normal aging, and can contribute to neurotoxicity through a variety of mechanisms including the production of free radicals leading to oxidative stress, excitotoxicity and by promoting inflammatory reactions. A growing body of evidence similarly supports a deleterious role of iron in the pathogenesis of TBI. Iron deposition in the injured brain can occur via hemorrhage/microhemorrhages (heme-bound iron) or independently as labile iron (non-heme bound), which is considered to be more damaging to the brain. This review focusses on the role of iron in potentiating neurodegeneration in TBI, with insight into the intersection with neurodegenerative conditions. An important implication of this work is the potential for therapeutic approaches that target iron to attenuate the neuropathology/phenotype related to TBI and to also reduce the associated risk of developing neurodegenerative disease.

## Introduction

Traumatic brain injury (TBI) is a leading cause of death and disability worldwide, particularly amongst young adults. Ten million individuals are affected by TBI annually, costing a staggering $9–10 billion/year (Gardner et al., 2017). The aged population have a greater risk of sustaining a TBI, with frequent falls being the major cause of injury, and they also have worse outcomes post-injury compared to other age groups (Stocchetti et al., 2012). Aging is also accompanied by a number of co-morbidities which may contribute to poorer outcomes in these individuals following TBI (Stocchetti et al., 2012). Common secondary events that follow the primary impact are neuronal cell death, oxidative stress, brain oedema, blood-brain barrier (BBB) breakdown, and inflammation (Toklu and Tumer, 2015). TBI often results in debilitating long-term cognitive and motor impairments, and there are currently no approved treatments available for TBI patients (Bramlett and Dietrich, 2015). This highlights the need for therapeutic agents that can alleviate brain damage and the deficits caused by the primary injury and more specifically the reversible secondary pathologies that develop after TBI.

**FIGURE 1:**
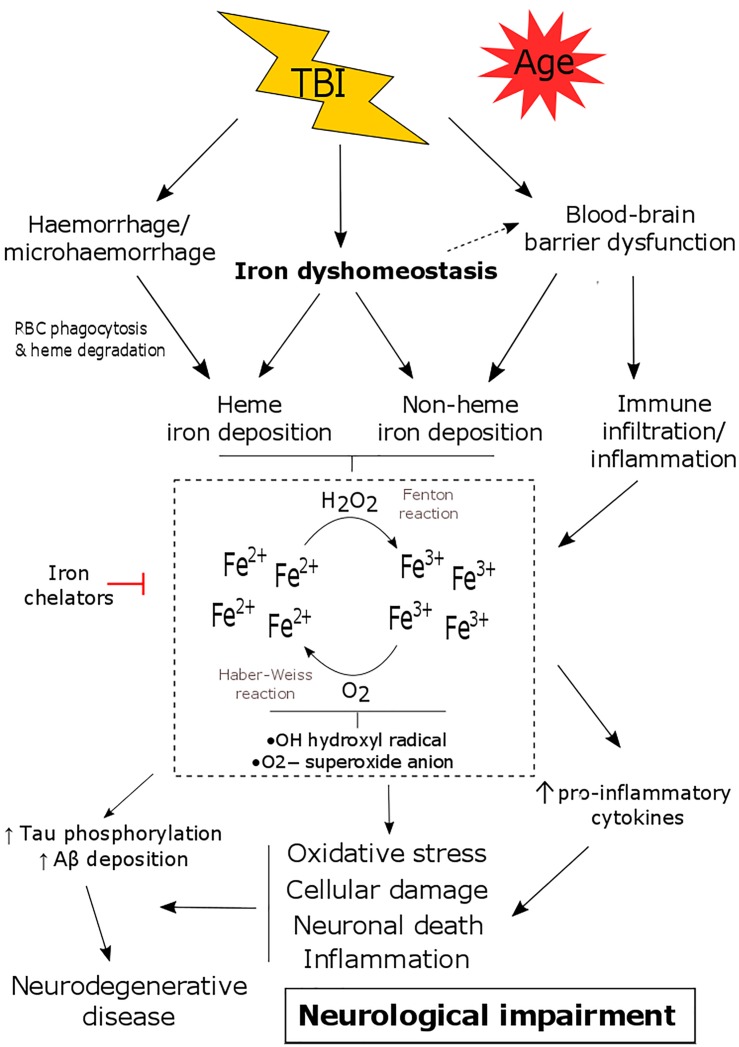
The consequence of iron dyshomeostasis following TBI. TBI results in several secondary events including blood-brain barrier (BBB) breakdown, hemorrhage and iron dyshomeostasis. Together this leads to the accumulation of heme and/or non-heme bound iron in the brain. Iron can participate in Haber-Weiss/Fenton reactions and can promote oxidative stress, neuronal death, inflammation as well as tau phosphorylation/amyloid-β deposition. This contributes to the pathology of TBI, ultimately resulting in neurological decline and an increased risk of developing neurodegenerative disease.

Iron homeostasis appears to be an important process in the pathobiology of TBI. Iron is essential for normal brain functioning where it acts as an essential cofactor for several enzymatic/cellular processes (Ke and Qian, 2007). However, impaired regulation of iron can result in the production of reactive oxygen species (ROS) and the consequent promotion of oxidative stress, which can wreak havoc on an already compromised brain in the context of TBI (Nunez et al., 2012). Interestingly, the accumulation of iron in various tissues and cells in the body and brain is an inevitable consequence of aging (Hagemeier et al., 2012; Del et al., 2015). A concomitant increase in the iron storage protein (i.e., ferritin), which can scavenge any excess iron and prevent undesired production of ROS, is also evident with aging (Andersen et al., 2014). However, failed or weakened antioxidant defenses and mitochondrial dysfunction that progresses with aging can disrupt the balance and allow for excessive iron to be released (Venkateshappa et al., 2012a,b; Andersen et al., 2014). This can cause pathological iron overload resulting in cellular damage that is considered to be a contributing factor in several degenerative diseases that are more prevalent with age, such as cancer, liver fibrosis, cardiovascular disease, diabetes (type II), and particularly neurological conditions such as Alzheimer’s disease (AD) (Smith et al., 1997; Kalinowski and Richardson, 2005; Ward et al., 2014; Hare et al., 2016). Abnormal brain iron deposition has also been discovered in other neurodegenerative diseases, such as Parkinson’s disease (PD) (Griffiths et al., 1999; Zhang et al., 2010; Barbosa et al., 2015), multiple sclerosis (MS) (Bergsland et al., 2017), amyotrophic lateral sclerosis (ALS) (Oshiro et al., 2011), Huntington’s disease (Agrawal et al., 2018), and Friedreich’s ataxia (Martelli and Puccio, 2014), and there is now increasing evidence of altered iron levels in TBI patients (Raz et al., 2011; Lu et al., 2015). This raises the interesting proposition of an intersection between aging, iron, TBI and neurodegenerative disease. Whilst the role of iron in TBI is not well known, the literature suggests that the levels of iron (and other metals such as zinc) are abnormally regulated following injury, and that the pharmacological targeting (e.g., using chelators or chaperones) of these metals may be beneficial in improving outcomes. Iron chelation therapy has been approved for decades for the treatment of iron overload conditions, and there is a recent heightened interest for their use in neurodegenerative diseases (Kalinowski and Richardson, 2005; Dusek et al., 2016). Here, we review the role of iron dyshomeostasis in TBI and gain a deeper understanding of its involvement in neurodegeneration as well as neuroinflammation (Figure 1). We further examine the potential benefit of utilizing iron chelation therapy with the hope of limiting iron-induced neurotoxicity in TBI.

## Iron Homeostasis and Its Role in Neurological Function

Iron is an important cofactor involved in a variety of bodily functions including cellular metabolism, energy production, cell growth and differentiation, and gene expression. It is primarily recognized for its ability to bind oxygen, contained within the hemoglobin complex, to transport oxygen throughout the body (Pantopoulos et al., 2012). Iron is also, however, fundamental for neuronal development, synaptic plasticity, neurotransmitter processing, and myelination (Ke and Qian, 2007; Ward et al., 2014). In the brain, iron, as well as other biometals, are found in numerous cell types including neurons, astrocytes, microglia, and most abundantly in oligodendrocytes (Burdo et al., 1999; Angelova and Brown, 2015). Astrocytes that are an integral part of the BBB have the ability to take up circulating iron and redistribute it to other cells in the CNS (Dringen et al., 2007). Iron is crucial for the maintenance of myelin, and therefore oligodendrocytes require a constant supply of iron (Connor and Menzies, 1996). Iron is also essential for optimal mitochondrial function (Urrutia et al., 2014), which is critical for neurons and other brain residing cells that have significant energy and metabolic requirements.

Iron homeostasis is carefully regulated in mammals by a plethora of proteins and enzymes produced in the liver such as hepcidin and ceruloplasmin, and other iron transporters (divalent metal transporter 1: DMT1, ferroportin, and transferrin) and their receptors (Ke and Qian, 2007). The expression levels of these proteins/receptors are in turn regulated by iron responsive elements (IRE) and iron regulatory proteins (IRP) especially when iron levels are low (Torben et al., 2007). Iron generally exists in an insoluble ferric (Fe^3+^) or soluble ferrous (Fe^2+^) state and is an active participant in electron transfer/redox reactions, which can drive oxidative stress-induced cellular toxicity (Graf et al., 1984; Nunez et al., 2012). Iron is solely absorbed through the diet, usually in its ferric form, and needs to be reduced to Fe^2+^ for its essential cellular uptake via transporter DMT1, with ferrous iron also released from cells via ferroportin (Gunshin et al., 1997; Donovan et al., 2000). In the circulation, iron is transported by binding to transferrin (ferric state; 2:1 ratio) whereas in tissues it is stored in a stable conformation by ferritin, a protein that consists of two subunits (i.e., ferritin heavy chain and light chain) (Ke and Qian, 2007; Nunez et al., 2012; Ward et al., 2014). The main regulator and inhibitor of iron release is hepcidin, which is secreted by the liver in response to stimulants (e.g., human hemochromatosis protein (HFE), inflammatory mediator IL-6, lipopolysaccharides (LPS) or high transferrin-Fe^3+^ complexes) ultimately to reduce levels of iron in the body (Ganz, 2003; Ward et al., 2014).

## The Metabolism of Iron in the Brain

**FIGURE 2:**
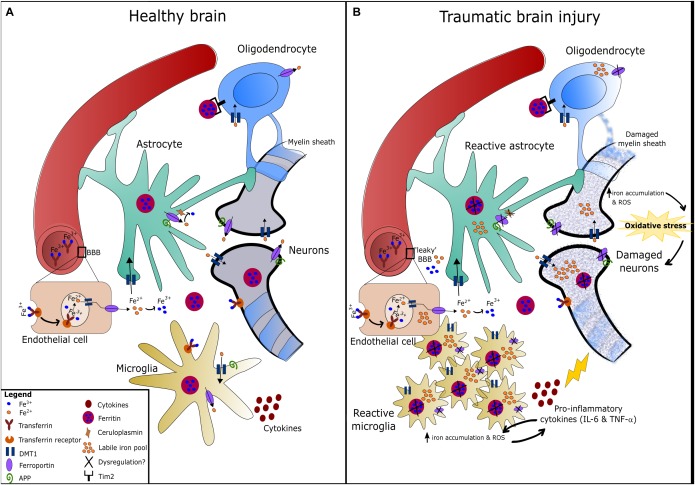
Iron metabolism in the healthy and traumatically injured brain. **(A)** Circulating iron (Fe^3+^) passes the blood-brain barrier (BBB) either bound to transferrin or independently, and is then taken up by endothelial cells expressing transferrin receptors via endocytosis. The acidic pH environment of the endosome detaches Fe^3+^ from transferrin and Fe^3+^ is then reduced to Fe^2+^ by ferric reductase (Ward et al., 2014). In the brain, Fe^2+^ is uptaken via DMT1 and distributed amongst astrocytes, neurons, oligodendrocytes, and microglia. Inside these cells, Fe^2+^ is converted to Fe^3+^ and stored by ferritin to prevent oxidative damage (Oshiro et al., 2011). Oligodendrocytes express Tim2 receptor that binds ferritin and iron can be imported through this mechanism, in addition to DMT1. Neurons and microglia can also uptake transferrin-bound iron via transferrin receptors (Ward et al., 2014). Ferrous iron is exported from cells via the iron exporter, ferroportin, which is present on all cell types. Fe^2+^ is rapidly oxidized to Fe^3+^ once outside the cell by ferroxidases such as hephaestin or ceruloplasmin (expressed on astrocytes mainly). Amyloid precursor protein (APP) is expressed on neurons, astrocytes and microglia, and has been found to facilitate iron export in neurons by stabilizing ferroportin (Wong et al., 2014). **(B)** Injury to the brain/neurovascular unit causes BBB damage, microglia activation, astrogliosis, and eventually damage to neurons and the myelin sheath surrounding axons. Dysregulation of iron metabolism in the brain following TBI can result in the accumulation of redox-active ferrous iron in various brain cells. This is possibly due to alterations in the expression/function of regulatory proteins such as ferroportin, ferritin and ceruloplasmin, which fail to export iron from cells and thereby increases the labile iron pool. Iron accumulation in microgia can cause increased pro-inflammatory cytokine production, and vice versa (Urrutia et al., 2014). Taken together, iron accumulation in the brain can promote oxidative stress that contributes to neurodegeneration.

The entry of iron into the brain parenchyma is restricted by the BBB due to its hydrophilic nature. Iron (ferric iron: Fe^3+^) can enter through endothelial cells that line the BBB typically via endocytosis of transferrin/transferrin receptors and to a lesser extent either independently (non-transferrin bound iron) or as a low molecular weight complex (Ke and Qian, 2007; Torben et al., 2007; Ward et al., 2014). Iron is released from transferrin due to the acidic pH environment of the endosome, is then reduced to ferrous iron and thus contributes to the labile ferrous iron pool (Ke and Qian, 2007). Ferroportin is the only known iron exporter for almost all cell types including neurons and astrocytes (Donovan et al., 2000). Ceruloplasmin, which is primarily a copper containing α2-globulin (binds six copper ions) and mainly present as a GPI-linked membrane protein in the brain, is also an important regulator of iron-mediated transport via binding ferroportin and driving the conversion of Fe^2+^ to Fe^3+^ by its ferroxidase activity (Attieh et al., 1999). Amyloid precursor protein (APP), a key protein in AD pathology, stabilizes ferroportin to facilitate iron export in neurons, and its activity has recently been found to be regulated by iron (Rogers et al., 2008; Wong et al., 2014). Ceruloplasmin and APP expression are both increased alongside iron accumulation in the brain of TBI patients and brain injured mice, and were found to elicit neuroprotective effects (Ayton et al., 2014). Figure 2 summarizes the proteins/receptors involved in iron metabolism in the brain.

Since iron is a transition metal, Fe^2+^ and Fe^3+^ can react with hydrogen peroxide and oxygen, respectively, catalyzing hydroxyl and superoxide radicals and can also react with lipids leading to the formation of alkoxy and peroxy radicals (Gutteridge, 1982). This is known as the Haber-Weiss and Fenton reaction. Hence, iron can increase ROS by participating in redox reactions and in turn lead to cellular oxidative stress and eventually cell death (Ke and Qian, 2007). Sufficient iron storage in the brain either by ferritin, neuromelanin (mainly in substantia nigra neurons) or hemosiderin is essential to prevent oxidative damage (Angelova and Brown, 2015).

Deficiency of iron or excessive iron, or changes in iron-regulating protein expression, can therefore be catastrophic to cells/tissues and are known to contribute to numerous disorders such as anemia, aceruloplasminemia, neuroferritinopathy, mitochondrial membrane protein-associated neurodegeneration (MPAN) and pantothenate kinase-associated neurodegeneration (PKAN) (Levi and Finazzi, 2014; Hogarth, 2015). Patients with inherited conditions that develop increased brain iron accumulation present with cognitive, motor and neuropsychiatric decline (Schröder et al., 2012; Hogarth, 2015). Excessive iron accumulation is harmful because it can promote the formation of free radicals resulting in oxidative stress, lipid peroxidation, protein aggregation and eventually cell/neuronal death (Nunez et al., 2012). On the other hand, iron deficiency can result in impaired myelin generation, neurotransmission and enzymatic processing. Iron deficiency anemia has been linked to the development of AD, and individuals with anemia are twice as likely to develop mild cognitive impairments over time (Faux et al., 2014). A recent study consisting of 939 patients with TBI found that anemia (i.e., presenting with low hemoglobin levels and low plasma iron), which affects approximately half of hospitalized TBI patients, is associated with poor outcomes following TBI (Litofsky et al., 2016). Taken together this shows that a delicate balance of iron metabolism needs to be achieved to protect the brain against its potentially harmful effects, in both healthy and diseased states.

## Iron in Neurodegenerative Diseases

Iron dyshomeostasis appears to be a central factor in neurodegenerative conditions such as AD, PD, ALS, Huntington’s disease, and Friedreich’s ataxia (Oshiro et al., 2011; Agrawal et al., 2018). For decades, deposits of iron have been detected in brain lesions of patients with these neurodegenerative conditions (Hagemeier et al., 2012; Barbosa et al., 2015; Adlard and Bush, 2018). How iron accumulates in the diseased/injured brain is not known, however, heme-bound iron that is derived from the circulation and non-heme bound iron (i.e., all iron not bound to heme protein) are both present in affected tissue (Nisenbaum et al., 2014; Ward et al., 2014). It is likely that the deposition of iron into the brain parenchyma in these conditions is the result of either hemorrhage/microbleeds (i.e., heme-bound iron), damaged cells (i.e., neurons, oligodendrocytes and microglia), and myelin, inflammatory processes, degradation of erythrocytes and heme proteins, and/or dysregulation of important iron-related proteins (e.g., ceruloplasmin, ferroportin) (Hametner et al., 2013; Hare et al., 2013; Nisenbaum et al., 2014). Excessive iron that is localized in affected neurons and glia can be found in brain regions such as the substantia nigra (dopaminergic neurons) in PD (Barbosa et al., 2015), and can also accompany regions of amyloid plaques and neurofibrillary tangles in AD (Smith et al., 1997). Similarly, patients with MS present with increased iron deposition in deep gray matter regions correlating with poor integrity of the white matter tissue (Bergsland et al., 2017). Increased brain iron accumulation in these neurological conditions have correlated with poorer functional outcomes and disease severity in patients (Zhang et al., 2010; van Duijn et al., 2017).

Although it is not yet clear whether iron is a causative or a contributing factor, its presence drives redox reactions, inflammatory processes as well as mitochondrial dysfunction (Urrutia et al., 2014). Iron accumulation in dopaminergic neurons, which are typically affected in PD, can exacerbate α-synuclein pathology and induce autophagy dysfunction (Wan et al., 2017). Ferric iron (Fe^3+^) can cause the formation of neurofibrillary tangles by binding to aggregated hyperphosphorylated tau proteins and can also become reduced to its destructive redox-active form (free Fe^2+^) during the process (Yamamoto et al., 2002; Oshiro et al., 2011). Iron can promote the toxicity of amyloid beta (Aß) by affecting aggregation, oligomerization, and amyloidosis of the peptide (Liu et al., 2011). It is also likely that the pathogenicity of Aß (e.g., in AD) is at least in part due to its interaction with iron and the potentiation of redox reactions (Rottkamp et al., 2001). In addition, expression of proteins that are involved in the regulation of iron are altered in these disease states; ceruloplasmin, ferroportin, and furin (regulates hepcidin) are decreased while DMT1 (iron importer) and transferrin are increased (Hare et al., 2013; Ward et al., 2014; Peters et al., 2015). The main iron storage protein in the brain, ferritin, is elevated in the cerebrospinal fluid (CSF) of patients with AD as well as areas containing amyloid plaques, and this protein also increases with age (Jellinger et al., 1990; Zecca et al., 2001; Ayton et al., 2018). These regulatory proteins in addition to iron are also associated with the production of Aß and hyperphosphorylated tau (Rogers et al., 2008). Together this reveals that the failure lies with various crucial proteins involved in the regulation of iron and is a possible explanation for the accumulation of iron in the brain.

Overall, increased iron accumulation in the brain, over and above the age-related changes, may be an indirect indicator of the progression and severity of AD and PD, and possibly other neurological conditions (van Duijn et al., 2017; Adlard and Bush, 2018). Elevated brain iron as measured by quantitative susceptibility mapping (via MRI) and increased CSF ferritin levels observed in patients with AD and mild cognitive impairment, were found to be predictive of cognitive decline over the long-term, and correlated with amyloid-β pathology (Ayton et al., 2017) and apolipoprotein E genotype (Ayton et al., 2015), respectively. Considering that brain iron content increases with age and that age is a major risk factor associated with neurodegenerative disease, the detection of iron using non-invasive imaging techniques may therefore prove to be a useful diagnostic tool as an early indicator of disease onset.

## Interplay of Iron, Inflammation and Neurodegeneration

The immune system similarly plays an important role in the pathogenesis of neurodegenerative diseases although its interaction with biometals is unappreciated. Immune cells are also equipped with transferrin and ferroportin receptors to enable the influx/efflux of iron to meet their cellular needs. In particular, phagocytic immune cells such as microglia (brain-specific macrophages) have an important role in the regulation and distribution of iron since they function to engulf and recycle damaged/dying cells and thereby release iron into the brain interstitial space (Moos, 2002; Andersen et al., 2014). Intracellular iron accumulation is associated with macrophage infiltration/polarization and increased TNF-α expression (Kroner et al., 2014). Indeed, inflammatory cytokines (mainly IL-6 and TNF-α) and mediators such as LPS induce the expression of iron transporter receptors (e.g., DMT-1) and promote an increase in the accumulation of iron in neurons and microglia (Urrutia et al., 2014).

Iron can accumulate in myeloid cells including microglia and monocytes, causing oxidative damage to the cell and promoting chronic neuroinflammation. This is a typical feature observed in several neurological conditions including MS and AD (Andersen et al., 2014; Stuber et al., 2016). *In vitro* studies reveal that iron can directly increase NFκB activity and stimulate pro-inflammatory cytokine production in microglia (Saleppico et al., 1996). Indeed, hemosiderin-laden macrophages were present in the frontal and temporal lobes of a patient with mild TBI (Bigler, 2004). Damaged oligodendrocytes and myelin in neuroinflammatory conditions including MS were determined as the main sources of extracellular iron in the brain parenchyma (Hametner et al., 2013).

This interplay of iron deposition with phagocytic cells (macrophages) can lead to neuronal damage not only due to oxidative stress and free radical formation but also through the promotion of pro-inflammatory mediators that further contributes to secondary damage in TBI. There is also the hypothesis that the inflammatory cells that migrate to the site of injury in TBI (or plaque regions in neurodegenerative diseases) are largely responsible for depositing iron (Sastry and Arendash, 1995; Andersen et al., 2014). Hence, an unrecognized link between iron accumulation and the involvement of the immune response in TBI warrants investigation considering both these processes are persistently active in the brain from weeks to months following injury. Whether iron dyshomeostasis in the brain is the process that promotes glial reactivity and drives neuroinflammatory responses, or vice versa, is unknown. Perhaps both these processes are simultaneously contributing to brain damage and consequential neurological impairment following TBI. Supporting this, a recent review proposed a positive feedback cycle in which mitochondrial dysfunction, inflammation, and iron accumulation are critical synergistic mechanisms that induce and enhance one another, and eventually become responsible for neuronal/cell death in the pathogenesis of neurodegenerative diseases (Nunez et al., 2012; Urrutia et al., 2014).

## The Role of Iron in TBI

Iron deposits that accumulate in the brain can consist of both non-heme and heme iron sub-types. These features are more commonly observed in neurodegenerative conditions such as AD, although increasing evidence suggests that both these subtypes of iron are increased in the injured brain after TBI (Nisenbaum et al., 2014). Heme-bound iron is commonly found coinciding with intracranial hemorrhage, along with deposition of hemosiderin and ferritin due to the phagocytosis of erythrocytes by microglia/macrophages (Koeppen et al., 2008; Nisenbaum et al., 2014).

A feature seen in TBI as well as several neurodegenerative diseases is the formation of cerebral microhemorrhages. These have been observed at both the acute and chronic stages of TBI, in experimental models of single and repetitive TBI (Donovan et al., 2012; Glushakova et al., 2014) as well as in a subgroup of mild TBI patients (Park et al., 2009; Hasiloglu et al., 2011; Nisenbaum et al., 2014). The clinical relevance of cerebral microhemorrhages on TBI outcome is not yet clear (Scheid et al., 2006; Irimia et al., 2018; van der Horn et al., 2018) although the presence of microhemorrhages following TBI is a suggested predictor of injury severity (Huang et al., 2015; Lawrence et al., 2017). Microbleeds have been negatively associated with cognitive outcomes in patients with mild cognitive impairment, stroke and MS (Werring et al., 2004; Zivadinov et al., 2016; Li X. et al., 2017). Microhemorrhages are interestingly associated with areas of white matter damage, BBB breakdown, demyelination, and inflammation in experimental TBI (Glushakova et al., 2014). Microbleeds, which can be detected by Prussian blue iron staining, reveal an abnormal accumulation of iron and ferritin/hemosiderin that have been found to be toxic to brain-specific cells (neurons, astrocytes, and microglia) and endothelial cells (Glushakova et al., 2014). More importantly, non-heme bound iron and free iron are also elevated in the brain following TBI and are considered more problematic (Nisenbaum et al., 2014), as shown in Figure 2.

The origin of brain iron deposits independent of heme-bound iron in TBI is not yet known although several hypotheses exist. Dysregulation of ceruloplasmin (e.g., decreased levels) can lead to an increase in free copper ions and a concomitant decrease in ferroxidase activity, thereby reducing clearance of iron from the CNS (i.e., increasing iron deposition). Ceruloplasmin deficient mice subjected to TBI showed elevated brain iron levels and exacerbated infarct volume (Ayton et al., 2014). TBI (and specifically intracerebral hemorrhage: ICH) often accompanies hemorrhage, increased vascularisation and BBB breakdown, which likely results in heme-bound iron deposition in the brain initially. In this instance, the enzyme heme-oxygenase 1 (HO-1) is involved in the degradation of heme-bound iron into free iron or non-heme bound iron (Beschorner et al., 2000). HO-1 is also suggested to be involved in induction of antioxidants bilirubin and biliverdin, and can therefore protect against oxidative stress. Indeed, HO-1 expression is heightened following TBI in both experimental models (Fukuda et al., 1995; Okubo et al., 2013) and in humans over a long-term period (Beschorner et al., 2000). HO-1 is likely protective at the acute stage through its antioxidant properties and clearance of heme-bound iron but it also could be responsible for non-heme bound iron accumulation in the brain, thereby increasing labile iron content over time. Free iron released from the degradation of heme proteins is a main source of oxidative stress, particularly in the injured brain (Chang et al., 2005). Excessive quantities of labile iron (Figure 2) can be harmful by inducing excitotoxic-related neuronal death, impaired neurotransmission, and mitochondrial dysfunction (Nunez et al., 2012; Urrutia et al., 2014; Ward et al., 2014).

Literature characterizing the metal profile and spatiotemporal distribution of iron is relatively sparse in TBI of varying injury severities and repetitive injuries, as well as ICH and stroke, in both clinical (see Table 1) and animal studies (see Table 2). Importantly, the consequence of iron accumulation in the brain following TBI is currently unknown, although it is associated with the development of post-traumatic epilepsy (Willmore et al., 1978; Willmore and Ueda, 2009; Ueda et al., 2013). Brain-region specific accumulation of iron has been documented in patients with mild TBI, via magnetic resonance imaging (MRI) approaches such as Magnetic field correlation (MFC) and susceptibility weighted imaging (SWI) (Raz et al., 2011; Nisenbaum et al., 2014; Lu et al., 2015). Various regions of the brain are affected including the hippocampus, globus pallidus, thalamus, substantia nigra, and corpus callosum, as summarized in Table 1. Non-heme iron is more sensitively detected by MR imaging techniques although limitations do exist (e.g., tissue fluid content can affect iron quantitation) (Raz et al., 2011). However, it can be concluded that non-heme bound iron accumulates in deep gray matter areas of the brain and may contribute to pathological secondary injury (e.g., oxidative stress) following TBI since abnormal iron deposition correlated with poor cognitive outcome (Raz et al., 2011; Lu et al., 2015).

**Table 1:** Brain iron accumulation in brain injury; evidence from clinical studies.

Brain injury	Patients	Detection method	Iron deposits/ region	Associated pathology	Outcome/prognosis	Study
Stroke	Ischemic stroke (*n* = 77) and transient ischemic attack (*n* = 74)	SWI	Lenticular nucleus	Correlated with cerebral microbleeds	N/A	Liu et al., 2016
	
	Unilateral ischemic stroke (*n* = 36)	T2-MRI	Thalamus	N/A	N/A	van Etten et al., 2015
	
	Ischemic stroke patients (*n* = 172)	R2 mapping, MRI	Thalamus, ipsilateral to infarct location	N/A	Associated with poor functional and cognitive outcome, and depression/anxiety at 1 year	Kuchcinski et al., 2017

ICH	Spontaneous ICH patients (*n* = 2, case study)	T2-MRI	Perihematoma, basal ganglia region (at 7 days)	N/A	N/A	Chaudhary et al., 2016

TBI	Mild TBI patients (*n* = 39)	SWI	Caudate nucleus, lenticular nucleus, hippocampus, thalamus, right substantia nigra, red nucleus, and the splenium of the corpus callosum	N/A	Negative correlation with Mini-Mental State Examination (MMSE) scores in mild TBI group compared to controls	Lu et al., 2015
	
	Mild TBI patients (*n* = 28)	MRI mapping/ MFC	Globus pallidus and thalamus	N/A	High MFC values (suggesting iron deposits) inversely correlated with cognitive function (i.e., Stroop color word test)	Raz et al., 2011
	
	TBI patients (*n* = 19)	PCR and IHC	Peri-contusional cortex (12 and 48 h post-injury)	Increased ferritin H-chain expression in neurons and iron in glial cells	N/A	Liu et al., 2013

Repetitive mild TBI/ CTE	CTE (Dementia Pugilistica)	Laser micro-probe mass analysis	Temporal cortex	Iron deposits in neuronal neurofibrillary tangles	More prominent in Dementia Pugilistica than AD	Bouras et al., 1997


*CTE, chronic traumatic encephalopathy; IHC, immunohistochemistry; ICH, intracerebral hemorrhage; MRI, magnetic resonance imaging; MFC, magnetic field correlation; MMSE, Mini-Mental State Examination; N/A, not available; PCR, polymerase chain reaction; SWI, susceptibility weighted imaging.*

**Table 2:** Brain iron accumulation in brain injury; evidence from experimental studies.

Brain injury	Model	Iron deposits/ region	Time-period	Associated pathology	Outcome/ prognosis	Study
Stroke	Rat, middle cerebral artery occlusion (MCAO)	Thalamus; increased iron in microglia (at 3 wks), parenchyma (at 7 wks) and APP deposits (at 24 wks)	Over 24 weeks post-injury	Neuronal loss, increased microglia activation and HO-1 expression	Behavioral deficits up to 24 wks	Justicia et al., 2008
	
	Rat, permanent photo-thrombotic cortical vessel occlusion	Free (labile) iron and total iron increased in the ischemic lesion	1 h post-injury	N/A	Iron chelator 2,2’-dipyridyl (Bipyridine), injected 15 min or 1 h post-injury, improved BBB integrity	Millerot-Serrurot et al., 2008

ICH	Rat, ICH model – collagenase	Non-heme and total iron in injured striatum	3 days and 4 wks post-ICH	Increased lesion volume	N/A	Auriat et al., 2012
	
	Mice, ICH model – collagenase VII-S injected in the left striatum	Peri-hematomal region	Peaking at 3 days post-ICH	Increased lesion volume and neuronal degeneration	Neurological deficits up to 28 days. Iron chelator VK-28 reduced brain oedema, ROS and white matter injury yet promoted M2-microglia polarization and improved outcomes	Li Q. et al., 2017
	
	Rat, ICH, autologous whole blood injected into basal ganglia	Increased brain non-heme iron, transferrin/transferrin receptor and HO-1 levels	Over 4 weeks	N/A	N/A	Wu et al., 2003

TBI	Mice, CCI model	Ipsilateral parietal cortex (LA-ICPMS)	3–28 days post-CCI	N/A	N/A	Portbury et al., 2016; Portbury et al., 2017
	
	Mice, CCI model	Thalamus and internal capsule (T2-MRI)	1 and 2 months post-CCI	Reactive astrocytes and microglia	N/A	Onyszchuk et al., 2009

Repetitive mild TBI	Rat, mild CCI (2 impacts; 1, 3 or 7 days apart)	Extravascular iron deposits at lesion site (IHC)	14 days	Correlated with lesion volume and reactive glial cells	Behavioral and memory deficits at 1 month post-injury	Huang et al., 2013


*APP, amyloid precursor protein; HO-1, heme oxygenase-1; LA-ICPMS, laser ablation-inductively coupled plasma-mass spectrometry; MRI, magnetic resonance imaging; MCAO, middle cerebral artery occlusion.*

Recent studies (Portbury et al., 2016, 2017) thoroughly analyzed the time-dependent alteration in metals including iron, zinc, and copper, in the brain by laser ablation-inductively coupled plasma-mass spectrometry (LA-ICPMS) in a mouse controlled cortical impact (CCI) model. Elevated iron concentrations were detected at the impact site as early as 3 days and were persistently elevated up to 28 days in both young (Portbury et al., 2016) and aged mice (Portbury et al., 2017) following injury. Supporting this, histological staining revealed intracellular iron accumulated in the cortex and hippocampal regions of the injured hemisphere (Portbury et al., 2016). Another study subjected aged mice (21–24 month old) to the CCI model and found increased iron deposition using T2-weighted MRI in the ipsilateral thalamus that accompanied astrogliosis and microgliosis at 1 and 2 months post-TBI (Onyszchuk et al., 2009). The authors suggest that free iron may be elevated as a result of degenerating thalamic neurons (Onyszchuk et al., 2009). Therefore, the continued presence of both inflammation and iron accumulation in the lesion likely contributes to ongoing pathology following TBI, as summarized in Figures 1, 2.

## Iron in Aging, Injury and Neurodegeneration

It is well recognized that iron as well as iron-related proteins (e.g., ferritin and HO-1) progressively increase in the aging brain (Hirose et al., 2003; Stankiewicz et al., 2007). Microglia and astrocytes, but not oligodendrocytes, appear to accumulate iron more with age (Zecca et al., 2001; Angelova and Brown, 2015). Regions of the brain typically affected by age-related iron accumulation are the hippocampus, cortex, cerebellum, and most prominently the basal ganglia. Interestingly, these regions are commonly linked to neurodegenerative diseases (Angelova and Brown, 2015). Indeed, age remains one of the greatest risk factors associated with poor outcomes following TBI, and the development of neurodegenerative/neuroinflammatory conditions (Stocchetti et al., 2012; Ward et al., 2014). In aged individuals, iron accumulation in the brain could be caused by events that coincide with the aging process including increased permeability of the BBB, inflammation, dysregulation of iron-related proteins, mitochondrial dysfunction, and altered iron distribution within the CNS (Farrall and Wardlaw, 2009; Ward et al., 2014). Therefore, it is not clear whether age-related iron accumulation causes, or is rather a consequence of, neurodegeneration that commonly occurs with aging. Importantly, iron deposition in the aged brain has been linked to cognitive and motor deficits in the elderly (Pujol et al., 1992), and motor dysfunction in rhesus monkeys (Cass et al., 2007). Since aged individuals generally exhibit co-morbidities and present with systemic inflammation and altered neurovascular unit integrity among other age-related pathological processes (Stocchetti et al., 2012), this renders the brain vulnerable to iron-mediated oxidative damage (Stankiewicz et al., 2007). This can be particularly problematic for an already compromised brain following TBI. The extent of iron deposition between adults and elderly TBI patients has not yet been directly examined clinically. However, a recent study showed that aged (24 month old) mice have higher levels of iron (as well as other metals) in both the injured and contralateral (uninjured) hemisphere following TBI compared to young (3 month old) mice (Portbury et al., 2017). This heightened increase in brain iron levels can potentiate oxidative stress and neurotoxicity (as extensively covered in this review), and therefore is a possible explanation for the cognitive decline and overall worse outcomes observed in the aging TBI population. Further research is needed to better understand the role of iron in aging and its link to TBI and neurodegenerative disease.

## Therapeutic Relevance and Future Directions

Iron-mediated brain damage occurs in patients with TBI over a chronic time-period, and it is speculated that increasing iron deposition (with age) can predispose these individuals to the development of neurodegenerative diseases later in life or to post-traumatic epilepsy (see Figure 1). Hence, a targeted therapeutic approach to regulate iron levels in TBI patients will be an important advancement in limiting progressive neurodegeneration and neurological deficits that accompanies TBI of varying severities, and thereby reduce the risk of neurological disorders. Iron chelation therapies work by binding free iron and removing excess iron from the blood. Iron chelation not only works by scavenging labile free iron but some can also act as an antioxidant that neutralizes the radical reaction by donating hydrogen (e.g., deferiprone, HBED iron chelator) (Khalaf et al., 2018). They can also bind/remove other metals yet have a higher affinity for iron, and the absorption and tissue penetration properties of the different iron chelators can vary (e.g., desferrioxamine is poorly absorbed in the gut while deferiprone and deferasirox are orally active) thereby different routes of administration are needed (Kalinowski and Richardson, 2005; Dusek et al., 2016). Iron chelation therapy has only been investigated in a few pilot clinical trials of common neurological conditions including AD (Crapper McLachlan et al., 1991), PD (Devos et al., 2014; Martin-Bastida et al., 2017), and MS (Lynch et al., 1996). As yet, no compelling evidence has come to light with the use of iron chelators in these conditions, although they have proved relatively effective in attenuating disease progression in PD. The brain permeable iron chelator, deferiprone, decreased iron deposits in the substantia nigra, and slowed disease-severity progression in a small-scale study involving PD patients (*n* = 40) (Devos et al., 2014). However, another pilot clinical trial assessing deferiprone in PD patients only found a trending improvement in motor function over 6 months (Martin-Bastida et al., 2017); hence more extensive clinical and experimental studies are warranted. Baicalin and deferoxamine, which are iron chelating drugs that can (baicalin) or cannot (deferoxamine) pass through the BBB, were assessed in a rat model of PD. Both these treatments inhibited iron deposition in the substantia nigra as well as the striatum, dentate gyrus, dentate-interpositus, and cerebellum, protected dopaminergic neurons from death (Xiong et al., 2012).

This strategy of chelating iron has yet to be trialed in patients with TBI, or chronic traumatic encephalopathy (CTE) patients with repetitive head injury, nor has it been greatly explored in animal models of TBI. However, it remains a potential treatment option based on the few promising experimental TBI studies that have assessed the effect of iron chelators (i.e., deferoxamine or dextran-coupled deferoxamine) in experimental models that will be described hereafter.

## Iron Chelation in TBI

Only a handful of studies to date have investigated the therapeutic potential of chelating iron in experimental TBI models, mainly via the ferric iron chelator deferoxamine, as summarized in Table 3. However, deferoxamine has several limitations including its inability to cross the BBB, rapid elimination rate and it can theoretically generate superoxide radicals through binding Fe^3+^ leading to Fe^2+^ oxidation (Klebanoff et al., 1989; Hall et al., 2010).

**Table 3:** The effectiveness of iron chelators in experimental TBI models.

Severity	Model	Iron chelator	Effect of treatment	Time-frame (post-injury)	Protective?	Study
Mild TBI	Mice, CCI model	HBED (N,N′-Di(2-hydroxybenzyl) ethylenediamine-N,N′-diacetic acid monohydrochloride)	Improved motor function and neurological deficits. Reduction in cortical injury volume, microgliosis and oxidative stress markers	3 days	Yes	Khalaf et al., 2018
Moderate TBI	Rats, CCI model	Deferoxamine	Improved spatial memory (Morris water maze) but no change in cortical tissue loss between deferoxamine and vehicle control	Up to 17 days	Yes, but only in spatial memory performance	Long et al., 1996
Moderate-severe TBI	Rats, fluid percussion injury model	Deferoxamine	Reduced hydrocephalus development after TBI and decreased heme oxygenase-1 expression	24 h	No outcome measures tested	Zhao et al., 2014
Severe TBI	Rats, weight drop model	Deferoxamine	Improved Morris water maze performance (spatial memory) and reduced brain atrophy	28 days	Yes	Zhang et al., 2013
	Mice, weight drop model (loss of consciousness)	Dextran-coupled deferoxamine	Improved grip-strength compared to mice treated with deferoxamine or dextran alone	1 h	Yes	Panter et al., 1992


In a recent study, the brain permeable iron chelator HBED was assessed in its effectiveness to attenuate iron-mediated brain damage in a mouse model of TBI (i.e., CCI) (Khalaf et al., 2018). They found that HBED improved locomotor function along with a reduction in lesion volumes and hippocampal swelling. Interestingly, HBED also limited microgliosis and the expression of immune markers in the corpus callosum (Khalaf et al., 2018). This pointed to a potential mechanism through which the drug mediates its protective effects. However, a limitation of this study is that HBED treatment did not significantly reduce iron in the brain as measured by Perls’ staining (Khalaf et al., 2018). On the other hand, deferoxamine treatment reduced the levels of iron and iron-storage proteins (ferritin and transferrin) in the brain, which were elevated at 28 days post-TBI in rats. This was accompanied by a reduction in brain atrophy in the injured rats treated with deferoxamine as well as an improvement in spatial memory and learning, at 28 days after TBI (Zhang et al., 2013). A similar protective effect with deferoxamine treatment was also observed in earlier studies at an acute period following TBI (Panter et al., 1992; Long et al., 1996).

Several key limitations of these studies exist, including the short period of treatment, limited outcome measures and only one recent study assessed brain pathology at one time-point. Hence, there is a great need for long-term sequential analysis for the use of iron chelators (specifically those that are brain-permeable) in experimental TBI models, in which the influence of iron chelation on functional outcomes, pathology (e.g., oxidative stress, cell death, BBB breakdown), other biochemical alterations and also inflammation are essential before moving into clinical trials.

However, iron chelation therapy has been more thoroughly explored as a strategy for combating brain iron overload in ICH and to some degree in stroke (See Table 2), although these are still in the preclinical phase with only a few currently active clinical trials (Yeatts et al., 2013; Yu et al., 2015; Zeng et al., 2018). In particular, a small-scale clinical trial using deferoxamine in ICH patients (*n* = 21) showed improvement in oedema volume and hematoma absorption over a period of 30 days (Yu et al., 2015). Deferoxamine reduces odema, cell death, hippocampal degeneration and also inflammation following experimental ICH (Garton et al., 2016). Neuronal death and behavioral deficits were attenuated by deferoxamine treatment in aged rats with induced ICH (Hatakeyama et al., 2013). Deferiprone treatment has shown promise in reversing iron toxicity in the brain and improving outcome. Treatment was remarkably effective when deferiprone was combined with the antioxidant n-acetyl cysteine, in rats fed with a high iron-supplemented diet (Sripetchwandee et al., 2014, 2016). This combined treatment had a greater efficacy in improving learning and memory, long-term potentiation, mitochondrial function, BBB integrity and cell death, compared to individual treatment in iron-overloaded rats (Sripetchwandee et al., 2014, 2016). In addition, brain-related pathology including Aβ deposition, tau-hyperphosphorylation and dendritic spine loss were all significantly reduced to a greater extent with combined treatment (Sripetchwandee et al., 2016).

Most recently, the use of a ferroptosis (a form of iron-dependent cell death) inhibitor was shown to reduce iron deposition and result in a host of downstream benefits, including decreased neuronal degeneration and improved behavioral outcomes (motor and cognitive function) (Xie et al., 2018). Thus, ferroptosis may represent a new pathway of relevance for TBI, and such studies are currently underway in our lab.

## Conclusion

Brain iron accumulation is a common feature of most neurodegenerative/neurological conditions including TBI. It is increasingly becoming clear that iron plays a detrimental role in the pathogenesis of various neurodegenerative diseases, however, its relationship to TBI pathobiology still requires consolidation. Although iron chelation with deferoxamine has shown limited potential in experimental TBI models, using a chelator that can penetrate the BBB (e.g., deferiprone, HBED, VK-28), with a known ability to lessen iron-induced neurotoxicity in ICH and neurodegenerative diseases, could prove to be superior than previously tested iron chelators in improving outcome after TBI. Whether a specified time-window for treatment is required (e.g., immediate vs delayed treatment) also needs to be addressed (as does the notion that a combination of iron and zinc-targeted treatments may have a synergistic benefit in TBI). Targeting both iron accumulation and subsequent oxidative damage and/or inflammation by proposed combined therapy (e.g., other antioxidants or anti-inflammatory agents) has not yet been assessed preclinically in TBI (Hall et al., 2010). This approach has the potential of limiting acute secondary injury and protecting against neurological deterioration, as well as attenuating long-term neurodegeneration thereby reducing the risk of neurodegenerative disease development. Lastly, there is a great need for experimental studies to focus on the long-term consequences of iron-mediated damage considering iron deposition progresses over time; as seen with age, neurodegenerative diseases as well as TBI.

## Author Contributions

MD and PA conceived and constructed the manuscript.

## Conflict of Interest Statement

The authors declare that the research was conducted in the absence of any commercial or financial relationships that could be construed as a potential conflict of interest.
